# The Relationship Between Foot and Pelvic Alignment While Standing

**DOI:** 10.1515/hukin-2015-0037

**Published:** 2015-07-10

**Authors:** Sam Khamis, Gali Dar, Chava Peretz, Ziva Yizhar

**Affiliations:** 1 Gait and Motion Analysis Laboratory, Dana Children’s Hospital, Tel-Aviv Medical Center, Tel-Aviv, Israel.; 2 Department of Physical Therapy, Faculty of Social Welfare & Health studies, Haifa University, Mount Carmel, Haifa, Israel.; 3 Department of Physical Therapy, Sackler Faculty of Medicine, Tel-Aviv University, Ramat Aviv, Israel.

**Keywords:** coupling, feet, pronated, hyperpronation, mal-alignment, pelvis

## Abstract

A normal motion and segmental interrelationship has been determined as a significant factor in normal function. Yet, the relationship between distal segments and pelvic alignment needs further investigation. The aim of this study was to investigate the interrelationship between distal and proximal lower extremity segments while standing and during induced feet hyperpronation. Changes in alignment of the pelvis and lower extremities were measured at a gait laboratory using the VICON 612 computerized motion analysis system. Thirty-five healthy volunteer subjects were recruited. Four randomized repeated-measure standing modes were used: standing directly on the floor and then on three wedges angled at 10°, 15° and 20° to induce bilateral hyperpronation for 20 seconds. A significant (p<0.05) bi-variate relationship was found between the anterior pelvic tilt and thigh internal rotation, in all four standing positions (.41≤r≤.46, in all p<0.014). A combined effect of rotational alignment between segments and the cumulative effect of foot hyperpronation on pelvic tilt revealed that only the shank significantly affected pelvic alignment, acting as a mediator between a foot and a thigh with the thigh having a crude significant effect on the pelvis. When internal rotation of the shank occurs, calcaneal eversion couples with thigh internal rotation and anterior pelvic tilt. It can be concluded that in response to induced hyperpronation, the shank is a pivotal segment in postural adjustment.

## Introduction

A normal motion and segmental interrelationship has been determined as a significant factor in normal asymptomatic function. It has been suggested that abnormal movements and interaction between segmental alignments of the lower extremity increases risk of lower limb injuries. These abnormal movements have been described at different levels and in different planes such as foot hyperpronation (Churchill et al., 1998), ankle inversion (McClay, 2000), tibial internal rotation (Nigg et al., 1993), knee abduction and external rotation (Stefanyshyn et al., 2006).

Numerous studies have evaluated the relationship between foot position or movement and alignment of the lower extremity. Foot pronation is strongly coupled with internal rotation of the shank, whereas supination couples with external rotation, both in walking, running (Cornwall and McPoil, 1995; Inman, 1976; Stergiou and Bates, 1997), and standing (Khamis and Yizhar, 2007; Klingman et al., 1997). However, researchers still disagree as to the degree of involvement of these adjacent segments.

Pronation has also been associated with internal rotation of the hip (Khamis and Yizhar, 2007; Tibersio, 1997, 1998). Three-dimensional movement analyses have demonstrated opposite rotational movement between the tibia and femur during knee flexion/extension (Churchill et al., 1998; Iwaki et al., 2000; Wilson et al., 2000). Internal tibial rotation couples with external femoral rotation, and external tibial rotation with internal femoral rotation. However, in a standing position, unidirectional coupling occurs between the two segments (Khamis and Yizhar, 2007).

The relationship between hip-pelvic movements has been less documented. Notwithstanding the key interactions between adjacent segments, the interrelationship between distant segments may also be of great significance with respect to the symptom-free musculoskeletal function.

In a previously published study (Khamis and Yizhar, 2007), induced foot hyperpronation while standing was found to be coupled with the shank, thigh internal rotation and anterior pelvic tilt, confirming an interaction between the two planes of motion occurring between distant segments. A high correlation between adjacent segmental alignment was found in every two sequential modes. No correlation was found between the change in foot hyperpronation and pelvic response, therefore a weak direct relationship between foot and pelvic alignment was concluded.

The goal of this study was to evaluate the effect of the foot on the pelvis via the following mediators: a shank and a thigh. When evaluating and treating malalignment of the lower limb, it is essential to understand the mechanism responsible for the postural change. We hypothesized that induced hyperpronation initiates a segmental chain reaction, whereas the shank, thigh or both, play a significant role as mediators, thus leading to anterior pelvic tilt while standing. We examined the relationship between segments while in a natural standing position and while externally inducing alignment change.

## Material and Methods

### Setting and Subjects

The study was conducted at a gait and motion laboratory. After initial screening and a thorough musculoskeletal evaluation (by an experienced examiner), a convenience sample consisting of 35 healthy subjects (15 males and 20 females) were recruited. The age of the participants was 27.68 ±2.6 years, body mass 73.15 ±10.93 kg and body height 176.42 ±9.19 cm. Exclusion criteria were a history of musculoskeletal injuries, limited subtalar eversion (< 6.6 degrees) (Lattanza et al., 1988), abnormal foot alignment (pes planuvalgus or pes planuvarus) and leg length discrepancy (>5mm) which was measured by using the direct method, ASIS to medial malleolus and the indirect method using boards with known heights to level the pelvis (ten Brinke et al., 1999). The study was approved by the Tel-Aviv Sourasky Medical Center ethics committee. All subjects signed an informed consent form.

### Instrumentation

The VICON ® 612 computerized motion analysis system (8 cameras, 120 Hz, with a capturing volume of 3.5 × 3.5 × 2.5 m) was used to measure changes in pelvic alignment and lower extremities. A three dimensional biomechanical model (PlugInGait) by Vicon® was used, based on the work of Kadaba et al. (1990) and the Helen Hayes Hospital. Three retro-reflective markers were used to spatially identify the pelvis, thigh, shank and foot. Pelvic orientation was defined according to the lab’s zero reference point. Thigh rotation was defined as the thigh with respect to the pelvis; shank rotation as the shank with respect to the thigh. Eversion of the calcaneus, the coronal component of foot pronation movement was measured using a two dimensional algorithm based on the Root’s clinical model (Root et al., 1971), where the coronal angle is measured between the shank and the calcaneus bisections (Khamis and Yizhar, 2007) allowing uncomplicated clinical application of the results.

### Study Design and Procedures

Three wedges were arranged in the center of the lab’s capturing volume to induce hyperpronation. The wedges (58 by 58 cm), built from two equal slopes, were placed at 10, 15 and 20° tilting inward, connected in the center at their lowest point and covered with EVA (non slippery material) (Picture 1). Four standing modes were randomly captured (standing directly on the floor and on three individual wedges at different angles) for each subject. In all four modes, subjects stood for 20 s in a relaxed position using the same natural foot rotation alignment and the same base of support according to their pelvic width. Capturing began after 10 s, with each mode repeated 3 times. Subjects were repositioned between trials. The researchers were aware that placing the foot in a natural foot rotation alignment would produce an angle between the longitudinal foot axis and the sagittal plane, resulting in foot pronation in addition to the effect of the wedge. This change was homogenous in all standing modes. The total resultant eversion angle was measured.

### Measures

A 4 s sample (from the 14th to the 17th s in every mode) was taken for further analysis. Standing in a natural position was used as a baseline comparison. The 4 s average was calculated for each trial. The maximum calcaneal eversion angle in the coronal plane; shank and thigh rotation angle in the transverse plane; and pelvic tilt angle in the sagittal plane were chosen. Computer output included graphic plot angles with respect to time, supported by commercial spreadsheet software.

### Statistical Analysis

Statistical analysis was performed for the left side only. Bi-variate associations were estimated using Pearson’s correlation. Mixed effect models for repeated measures (proc mixed, SAS version 9.1) (Verbeke and Molenberghs 2001) were built to assess the segment with the most powerful effect on pelvic alignment, and determine whether the thigh or shank were intermediate factors between the foot and the pelvis (taking into account the various standing modes). Dependent variables were either the pelvic anterior tilt or thigh rotation alignment angles; independent variables were the modes (a fixed factor with 4 categories) and different combinations of the foot eversion, shank and thigh rotation alignment angles (covariates).

Mixed effect models were used for repeated measurements since the measurements in the 4 modes were related. Our aim was to determine whether the shank or thigh were mediators between the foot and pelvis. We built mediation models to identify a mechanism, and consider if the relationship between the foot and pelvis (via the inclusion of a third explanatory factor known as a mediator) was either shank, thigh or both. We assessed whether the foot would influence the shank, the thigh or both (mediators), which in turn would influence the pelvis. We looked for an indirect relationship rather than a direct relationship between the foot and pelvis. The intermediate segment between foot and pelvis (shank or thigh) is regarded as a mediator when it either increases or decreases the effect of the foot on the pelvis when adjusting to the given intermediate segment (specifically when comparing the crude and adjusted effects of the foot on the pelvis).

The following five models were built to evaluate the segmental interrelationship: 1. Foot, shank (two independent variables) and pelvis (dependent variable); 2. Foot, thigh (two independent variables) and pelvis (dependent variable); 3. Foot, shank (two independent variables) and thigh (dependent variable); 4. Shank, thigh (two independent variables) and pelvis (dependent variable); 5. Foot, shank, thigh (three independent variables) and pelvis (dependent variable).

All data analysis was performed by SAS version 9.1. A “crude effect” of a variant (unadjusted) related to the direct effect of a specific independent variable (foot, shank or thigh) on a dependent variable (thigh or pelvis) while simultaneously an adjusted effect related to the effect of this specific variable taking into account other variables. *p* values <.10 were considered non-significant; p values >.05 and <.10 were considered border line; *p* <.05 were considered significant.

## Results

Table 1 describes the changes in segmental alignment between modes. Most of the results indicate significant changes within segments. A significant cumulative change occurred when the slope’s angle was increased and reached a cumulative change of 7.05° on the left and 5.94° on the right in the calcaneal angle; 4.95° and 4.74° internal shank rotation on the left and right, respectively, and 2.91° and 4.21° internal thigh rotation on the left and right thigh, respectively. The average change in pelvic position reached 1.11° with 40% of the cases demonstrating a change of 2°–3° toward anterior pelvic tilt.

### Bi-variate Correlations between the Pelvis and the 3 Segments

The only significant (p<.05) relationship found when evaluating the bi-variate correlations between pelvic alignment and the 3 segments (thigh, shank and foot alignment) was between the pelvis and thigh alignment in all four standing modes (r=.42,.41,.43,.46; p=.01, .01, .01, <.001 respectively) (Table 2). Hence, the more the thigh internally rotated, the greater the anterior pelvic tilt angle. The relationship between foot and pelvic alignment was found to be non-significant. The foot and pelvic alignment relationship while standing directly on the floor, was found to be the worst (r= −.052, p=.76).

### Bi-variate Correlations between the Foot, Shank and thigh Alignments

No relationship was found between any two of the three segments in all four standing modes.

### Combined Effect of Rotational Alignment between Segments and the Cumulative Effect on Pelvic Tilt

#### A. Shank as a mediator between foot and pelvis taking into account standing conditions

**Figure 1:**
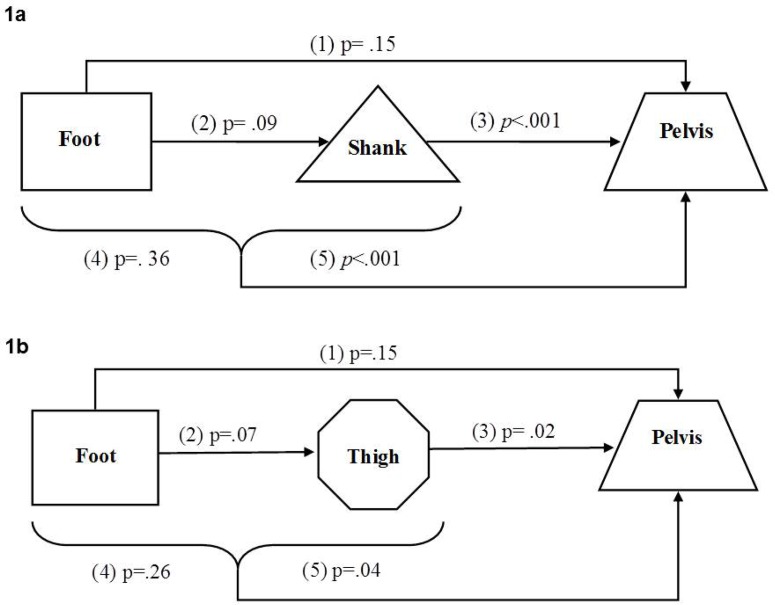
***1a.** A flow chart describing the combined effect of the rotational alignment between the foot and the pelvis mediated by the shank. A crude effect of the foot or shank relates to their individual direct effect on the pelvis, while simultaneously an adjusted effect relates to their effect on the pelvis taking into account one another. p value indicates significance of effect.*
Crude effect of the foot on the pelvis;Crude effect of the foot on the shank;Crude effect of the shank on the pelvis;Adjusted effect of the foot on the pelvis;Adjusted effect of the shank on the pelvis. ***1b.** A flow chart describing the combined effect of the rotational alignment between the foot and the pelvis mediated by the thigh. A crude effect of the foot or thigh relates to their individual direct effect on the pelvis, while simultaneously an adjusted effect relates to their effect on the pelvis taking into account one another. p value indicates significance of effect.*
Crude effect of the foot on the pelvis;Crude effect of the foot on the thigh;Crude effect of the thigh on the pelvis;Adjusted effect of the foot on the pelvis;Adjusted effect of the thigh on the pelvis.

Foot alignment had a non-significant crude and adjusted to shank effect on the pelvis (p=.15, p=.36, accordingly) (Figure 1a, Table 3). Shank alignment had a highly significant effect, both crude and adjusted to foot (p<.001) on the pelvis. However, the foot had a borderline effect on the shank (p=.09). Thus, we demonstrated in this model that the shank was not a mediator between foot and pelvis; since the crude effect of the foot on the pelvis, did not change and also remained non-significant when adjusting to the shank segment.

#### B. Thigh as a mediator between foot and pelvis taking into account standing conditions

Foot alignment had a non-significant crude and adjusted to thigh effect on pelvic alignment (p=.15, p=.26, accordingly) (Figure 1b, Table 3). Thigh alignment had a significant effect, both crude and adjusted to the shank (p=.02, p=.04, accordingly) on pelvic alignment. However, foot alignment had a borderline effect on thigh alignment (p=.07), thereby demonstrating that the thigh was not a mediator between the foot and the pelvis. The thigh was not considered a mediator, because the adjusted to thigh effect of the foot on the pelvis did not change and remained non-significant.

#### C. Shank as a mediator between the foot and the thigh taking into account standing conditions

**Figure 2:**
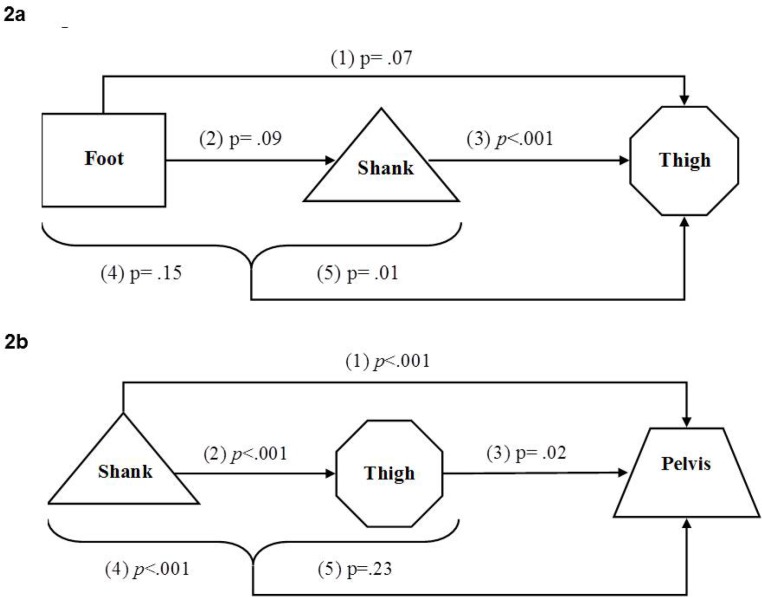
***2a.** A flow chart describing the combined effect of the rotational alignment between the foot and the thigh mediated by the shank. A crude effect of the foot or shank relates to their individual direct effect on the thigh, while simultaneously an adjusted effect relates to their effect on the thigh taking into account one another. p value indicates significance of effect.*
Crude effect of the foot on the thigh;Crude effect of the foot on the shank;Crude effect of the shank on the thigh;Adjusted effect of the foot on the thigh;Adjusted effect of the shank on the thigh. ***2b.** A flow chart describing the combined effect of the rotational alignment between the shank and the pelvis mediated by the thigh. A crude effect of the shank or thigh relates to their individual direct effect on the pelvis, while simultaneously an adjusted effect relates to their effect on the pelvis taking into account one another. p value indicates significance of effect.*
Crude effect of the shank on the pelvis;Crude effect of the shank on the thigh;Crude effect of the thigh on the pelvis;Adjusted effect of the shank on the pelvis;Adjusted effect of the thigh on the pelvis.

The crude and adjusted to shank effect of the foot on thigh were different (p=.07 borderline significant, p=.15 non-significant, respectively) (Figure 2a, Table 3). The shank was found to be a mediator since including it in the model altered the relationship between the foot and the thigh, thus decreasing the foot effect.

#### D. Thigh is a mediator between shank and pelvis taking into account standing conditions

The crude and adjusted to thigh effect of the shank on the pelvis were similar and significant (p<.001), therefore, the thigh was found not to be a mediator between shank and pelvis (Figure 2b, Table 3).

#### E. A multiple model was used to assess the combined effect of the 3 segments: thigh, shank and foot on the pelvis

**Figure 3:**
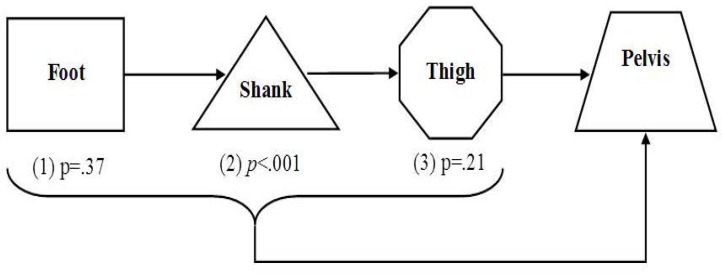
A flow chart describing the adjusted effect of the foot, shank and thigh on the pelvis taking into account one another. *p value indicates significance of effect.*
Adjusted effect of the foot on the pelvis;Adjusted effect of the shank on the pelvis;Adjusted effect of the thigh on the pelvis.

Figure 3 demonstrates the adjusted effect of each segment. Only the shank was found to have a significant effect on pelvic alignment (Table 3).

In view of previous results that the shank was determined to be a mediator between the foot and the thigh and that the thigh had a crude significant effect on the pelvis, a valid prediction model for pelvic alignment was found to be conditional only on the shank, which was found in all 4 standing modes (a non-significant interaction effect).

## Discussion

The goal of the present study was to examine the interrelationship between segments of the lower extremity while standing and during an induced postural change of hyperpronation. The main outcome was an anterior pelvic tilt occurring simultaneously with induced calcaneal eversion, through a mediating effect of the shank. The most interesting finding was that the shank segment was found to be the only segment significantly affecting thigh and pelvic alignment.

Examining the direct relationship between adjacent segments revealed a high bivariate correlation between thigh and pelvic alignment in all positions. Internal thigh rotation was coupled with anterior pelvic tilt. The relationship between these two segments has potential significance in terms of clinical evaluation and treatment: i.e. modification in pelvic alignment in the sagittal plane can be achieved by altering thigh alignment in the transverse plane.

When evaluating the cumulative effect of the segmental alignments, the calcaneal angle variation had no consistent significant effect on the shank, thigh and pelvic alignment. Furthermore, no significant effect of the calcaneal alignment on the shank was found while standing indicating a poor association between calcaneal eversion and internal tibial rotation, compared to the significant effect of foot alignment on the shank during walking and running (Cornwall and McPoil, 1995; Inman, 1976; Nigg et al., 1993; Stergiou and Bates, 1997). The degree of involvement between calcaneal eversion and shank internal rotation is indeed questionable. A number of researchers have found a high correlation between pronation and shank rotation while walking, suggesting measuring shank rotation as a reflection of subtalar movement (Cornwall and McPoil, 1995; Nigg et al., 1993). Others have also found the same unidirectional relationship but with a lower correlation (McClay, 2000; Nester, 2000; Reischl et al., 1999, van den Bogert et al., 1994). This discrepancy might be due to the versatile maneuvering of the foot (Kappel-Bargas et al., 1998) and the functional ability of the foot to compensate on different terrains. The extent of coupling between the rear foot and the shank is believed to be influenced by the orientation of the subtalar joint axis in the sagittal plane (DeLeo et al., 2004).

Several studies have found a wide variation of the direction of the subtalar joint axis that alters the correlation between the foot and the shank (Inman, 1976; Leardini et al., 2001; Sangeorzan, 1991). It has been suggested that subtalar joint axis orientation has a relatively greater effect on eversion motion compared to tibia motion in the transverse plane (Lundberg et al., 1989a,b,c). The relative motion between calcaneal eversion and tibial internal rotation while running also suggests a greater amount of calcaneal eversion compared to tibial internal rotation (ratio of 1.42–1.72) (McClay, 2000; Stacoff et al., 2000a,b,c). In addition, the absence of one unified biomechanical foot model can be a contributing factor to the varying results obtained.

Comparison of our results with data provided by other authors is difficult since no published data measuring or describing segmental coupling in standing has been found in the literature. Our results indicated that calcaneal alignment had a weak effect on the thigh and pelvis alignment while standing. However, calcaneal eversion couples with the proximal change of thigh internal rotation and anterior pelvic tilt, providing internal rotation of the shank.

We found that the shank was the mediator segment in adjusting posture, responding to the external intervention of hyperpronation and initiating a kinematic chain reaction. Of the three segments examined in the current study, the shank was found to have the most powerful effect on pelvic alignment. In 40% of our cases, a change of 2–3° was measured at the pelvis. We assumed this to be sufficient to cause functional changes, symptoms and limitations at the thigh, pelvis and lower back (McClay, 1997; Rothbart and Estabrook, 1998). Thus, changes in foot alignment may possibly lead to changes in the lumbar spine position, since some studies have found that the sagittal pelvic tilt and lumbar spine curvature seem to be highly correlated (Levine and Whittle, 1996).

These findings may reinforce the claim made by clinicians that foot alignment can be one of the sources of hip, pelvis or lower back signs and symptoms. We did not investigate this in the current study. Clinically, these results also support the importance of evaluating foot alignment while standing as an integral part of the evaluation and treatment of the pelvis and lower back. If foot alignment alternation is needed, a wedge can be used without a significant change in the alignment of the higher segments. However, if the goal in altering foot alignment is treating and adjusting more proximal segmental alignment, i.e. hip, pelvis and lower back, a significant change must occur in shank alignment. Measuring shank rotation might be suggested as a valid method for evaluating the effect on the proximal segments.

## Conclusions

A lateral wedge positioned under the foot significantly effects lower extremity alignment in a segmental chain. A modification in foot alignment leads to a change in the alignment of the lower extremity up to the pelvis. An interaction exists between nearby segments as well as between distant segments such as the shank and pelvis. This interaction in the normal population is significant and unidirectional. The shank plays an important role as a mediator between foot and pelvic alignment, on the condition that a change occurs in the shank alignment. For example, symptoms of the lower extremity and the lower back in the competitive athlete require a comprehensive biomechanical evaluation of the foot and shank and understanding of the interaction in order to assess the efficacy of treatment interventions such as foot orthotics or taping techniques.

## Figures and Tables

**Picture 1:**
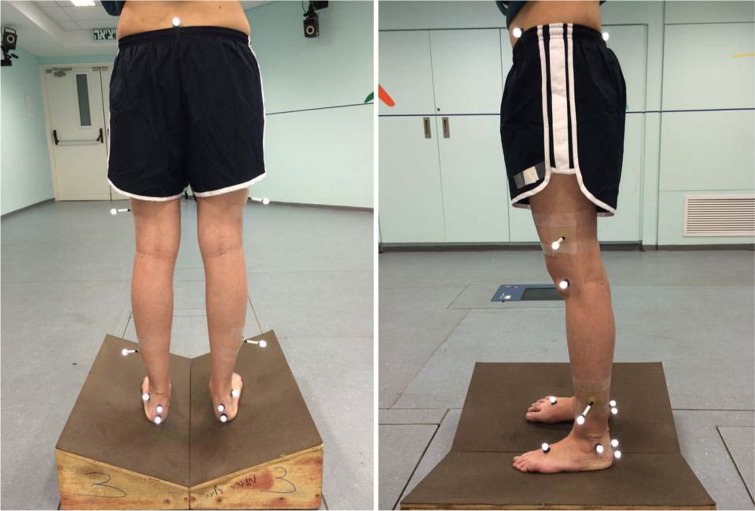
A subject standing on a wedge with applied markers while capturing data (left – posterior view, right – lateral view).

**Table 1:** Changes in segmental alignment (degrees) between modes

		**Left**		**Right**	
		Mean difference (SD Error Mean)	T (Sig.)^*^	Mean difference (SD Error Mean)	T (Sig.)^*^
	
**Calcaneal eversion angle**	W1-W0	2.59 (.55)	4.67 (<.001)^*^	1.18 (.59)	2.01 (.05)
	W2-W1	1.87 (.27)	6.81 (<.001)^*^	.54 (.49)	1.08 (.28)
	W3-W2	2.58 (.40)	6.35 (<.001)^*^	4.20 (.86)	4.86 (<.001)^*^
	W3-W0	7.05 (.95)	7.37 (<.001)^*^	5.9 (1.07)	5.54 (<.001)^*^
	
**Internal shank rotation angle**	W1-W0	2.35 (.26)	9.03 (<.001)^*^	2.44 (.27)	8.78 (<.001)^*^
	W2-W1	1.87 (.23)	8.05 (<.001)^*^	1.44 (.18)	7.95 (<.001)^*^
	W3-W2	0.72 (.23)	3.02 (<.001)^*^	0.85 (.25)	3.38 (<.001)^*^
	W3-W0	4.95 (.35)	13.97 (<.001)^*^	4.74 (.34)	13.64 (<.001)^*^
	
**Internal thigh rotation angle**	W1-W0	1.37 (.23)	5.78 (<.001)^*^	2.09 (.25)	8.23 (<.001)^*^
	W2-W1	0.67 (.20)	3.35 (<.001)^*^	1.38 (.19)	7.12 (<.001)^*^
	W3-W2	0.87 (.17)	4.9 (<.001)^*^	0.74 (.27)	2.75 (<.001)^*^
	W3-W0	2.91 (.25)	11.52 (<.001)^*^	4.21 (.39)	10.67 (<.001)^*^
	
		**Mean difference (Standard Error Mean)**		T (Sig.)^*^	
**Anterior pelvic tilt angle**	W1-W0	.51 (.51)		3.44 (<.001)^*^	
	W2-W1	.29 (.14)		2.07 (.04)^*^	
	W3-W2	.30 (.18)		1.66 (.10)	
	W3-W0	1.11 (.20)		5.52 (<.001)^*^	

W0: standing directly on the floor, W1: first wedge (10 degree angle): W2: second wedge (15 degree angle): W3: third wedge (20 degree angle); p<0.05

**Table 2:** Bivariate correlations between segments according to the standing mode

		**Left r (*p*)**
**Floor (0º)**	Foot-Shank	−0.138 (0.43)
	Shank-Thigh	−0.120 (0.49)
	Thigh- Pelvis	0.424 (0.01^*^)
	Foot - Pelvis	−0.052 (0.76)
**Wedge 1 (10º)**	Foot - Shank	0.155 (0.37)
	Shank-Thigh	0.064 0.71)
	Thigh- Pelvis	0.410 (0.01^*^)
	Foot - Pelvis	−0.138 (0.42)
**Wedge 2 (15º)**	Foot –Shank	0.171 (0.32)
	Shank-Thigh	0.070 (0.69)
	Thigh- Pelvis	0.429 (0.01^*^)
	Foot - Pelvis	−0.264 (0.12)
**Wedge 3 (20º)**	Foot -Shank	0 .238 (0.16)
	Shank-Thigh	0.049 (0.77)
	Thigh- Pelvis	0.458 (<0.001^**^)
	Foot - Pelvis	−0.231 (0.18)

* Correlation is significant at the 0.05 level (2-tailed).

** Correlation is significant at the 0.01 level (2-tailed).

**Table 3:** Estimated coefficients, significant effect and p of all the models. The number in bracket specifies the segments involved

**Figure**	**Effect**	**β**	**Significant effect**	**p**
1a	(1)	−0.039	0.027	0.15
	(2)	−0.076	0.045	0.09
	(3)	0.194	0.000	<0.001
	(4)	−0.023	0.023	0.36
	(5)	0.186	0.051	<0.001
1b	(1)	−0.039	0.027	0.153
	(2)	−0.062	0.035	0.077
	(3)	0.154	0.069	0.028
	(4)	−0.031	0.028	0.263
	(5)	0.142	0.070	0.045
2a	(1)	−0.061	0.034	0.07
	(2)	−0.076	0.045	0.09
	(3)	0.180	0.063	<.001
	(4)	−0.048	0.033	0.15
	(5)	0.166	0.064	0.01
2b	(1)	0.193	0.050	<.001
	(2)	0.180	0.063	<0.001
	(3)	0.154	0.069	0.02
	(4)	0.174	0.052	<0.001
	(5)	0.082	0.068	0.23
3	(1)	−0.020	0.026	0.43
	(2)	0.169	0.052	<0.001
	(3)	0.077	.0690	0.26
